# Biddle’s Review of Materia Medica

**Published:** 1852-05

**Authors:** 


### BIDDLE'S REVIEW OF MATERIA MEDICA.

This little work was prepared for the use of students, but the practitioner will find it convenient for reference. It is an admirable summary of Materia Medica, and to the student will prove a very useful handbook. Valuable as are the *United States Dispensatory* and *Pereira's Materia Medica*, they are far too voluminous to be used profitably as text-books. Dr. Biddle's "Review" is just such a manual as the student wants to recall the chief points of the lectures to which he has attended.

